# The New Age of Organ Donation—What Factors Have an Influence on the Attitude of Older People? An Attitudinal Survey in Southeastern Spain

**DOI:** 10.3390/ijerph19148524

**Published:** 2022-07-12

**Authors:** Beatriz Febrero, Javier Almela-Baeza, Inmaculada Ros-Madrid, José Alfonso Arias, Juan José Ruiz-Manzanera, María Isabel Jiménez-Mascuñán, Pablo Ramírez

**Affiliations:** 1 General Surgery Service, Virgen de la Arrixaca Hospital, 30120 Murcia, Spain; beatriz.febrero@um.es (B.F.); jjrmanzanera@hotmail.com (J.J.R.-M.); isjimiak@gmail.com (M.I.J.-M.); ramirezp@um.es (P.R.); 2 Department of Surgery, Pediatrics, Gynecology and Obstetrics, School of Medicine, University of Murcia, 30120 Murcia, Spain; inmaculada.ros3@um.es (I.R.-M.); josealfonsoariascanovas@gmail.com (J.A.A.); 3 Instituto Murciano de Investigaciones Biosanitaria IMIB-Arrixaca, 30120 Murcia, Spain; 4 Faculty of Communication and Documentation, University of Murcia, 30100 Murcia, Spain

**Keywords:** organ donation, attitudes, elderly, transplant, communication, social interaction

## Abstract

Currently, more than half of all donors are aged over 65 years, and previous studies have shown that this group is less willing to support organ donation. Objective: to analyse the attitude of people aged over 65 years toward organ donation and transplantation (ODT) and to determine how their psychosocial profile affects their attitude. Study population: citizens residing in southeastern Spain older than 65 years of age. A representative sample was obtained, which was stratified by gender and geographical location (n = 420). A validated questionnaire about ODT was used. Statistical analysis: a bivariate analysis was performed using the X2 test and a multivariate analysis. The favourable attitude toward the donation of one’s own organs was 53%. The psychosocial variables affecting attitude were mainly: having discussed ODT with one’s family (*p* < 0.001) or friends (OR 2.223), acceptance of cremation (OR 2.508), and acceptance of an autopsy (OR 2.578). Citizens aged over 65 tend to have an unfavourable attitude toward the donation of their own organs. The lack of dialogue about ODT in social and family settings, and the attitude to the manipulation of one’s own body after death, are aspects of a respondent’s psychosocial profile, which influence this attitude.

## 1. Introduction

Currently, citizens aged over 65 years account for a fourth of the Spanish population. What is more, future projections carried out by the Spanish National Institute of Statistics (INE) suggest that, by the year 2050, citizens who are older than 65 years will make up more than 30% of the population, comprising a total of nearly 13 million people.

It is clear that, because of the aging population, some approaches to the organ donation and transplantation (ODT) process have changed, making it necessary to include older patients on the transplant waiting list and assessing these older people as potential donors. At present, the average profile of a donor is someone aged 60.7 years, 6 years older than the mean age of a donor 10 years ago (54.6 years) [1,2]. In this context, several studies have shown that transplants from older donors have favourable results [3,4,5,6,7].

In this regard, older people make up an important subgroup of population given that this is the section of the population that provides the most organs to the transplant system. In spite of the high donation rates in our country in recent years [1], only a few studies have specifically analysed the attitude toward organ donation and the factors influencing their attitude, and the findings from these studies suggest that citizens of an older age are less willing to support organ donation [8,9]. Therefore, it would seem to be necessary to find out about their attitude toward ODT and the psychosocial variables affecting this attitude.

The objectives of this study were to analyse the attitude toward the donation of their own organs upon death and to investigate the psychosocial profile of citizens who are over 65 years in southeastern Spain, which can have an influence on their attitude.

## 2. Materials and Methods

### 2.1. Study Design

A cross-sectional study was carried out.

### 2.2. Study Population

The study population consisted of older people aged over 65 years residing in southeastern Spain (N = 322,811) [10]. 

### 2.3. Eligibility of the Study

Inclusion criteria: people over 65 years of age who gave their consent to participate in the study were included.

Exclusion criteria: people over 65 years of age with some kind of intellectual limitation that prevented them from understanding the study.

### 2.4. Sample Size

In order to carry out the calculation of the sample size, the 9 areas of the region were taken as a reference [11]. A representative sample was obtained, the sample was non-probabilistic, one-stage sampling. Individuals were selected according to sex quotas, proportional to the population by county (n = 420).

### 2.5. Measurement Instrument

A validated questionnaire about attitude toward ODT was used (Table 1) [12].

### 2.6. Fieldwork Process

The questionnaire was completed by three interviewers related to the Regional Transplant Coordination Centre during the period January to October 2018. The interviewers had previous experience in other studies of attitudes towards ODT in other groups with the application of questionnaires. In addition, before starting the study, the importance of the impartiality of the interviewer, reading the survey without interpretation, completing the survey in a quiet environment and without influence from other people was discussed with the interviewers. The interviewers approved their criteria for data collection after a pilot study of 50 interviews. The pretest was carried out in two health centres in two randomly selected areas on 50 people over 65 years of age, with an equal distribution of the sample according to the sex ratio in each area. Questionnaire completion was conducted orally for approximately 20 min, maintaining the respondents’ anonymity.

### 2.7. Variables Analysed

General variables: variables related to attitude toward organ donation; attitude toward the donation of your own organs upon death (including reasons for being in favour and against organ donation); variables of social and family interaction related to ODT (discussion about ODT with friends and family, knowledge of your partner’s opinion about ODT). The following variables were also analysed: knowledge of life support limitation and attitude toward controlled donation after circulatory death after life support limitation.

In order to analyse the aspects of the psychosocial profile of people over 65 years of age related to their attitude toward the donation of their own organs upon death, six categories were taken into account: (1) sociopersonal variables: geographical area, age, gender, and marital status; (2) variables of knowledge: knowledge of the concept of brain death (BD), knowing a donor or transplant patient, a belief that one might need a transplant organ at some point in time in the future, having received a talk about ODT, wanting to receive a talk about ODT; (3) variables of social and family interaction previously described; (4) variables of attitude toward the manipulation of the body upon death: acceptance of cremation, burial and autopsy, worry about scars or mutilation of the body after death; (5) variables of prosocial behaviour: being a blood donor, participating in prosocial activities; and (6) variables related to religion: religious attitude, belief about the opinion of one’s religion toward ODT.

### 2.8. Statistical Analysis

The data were analysed using the SPSS statistics program (version 24.0). A descriptive analysis was performed on the variables and a bivariate analysis using the chi square test. The variables with a statistically significant association were selected in order to carry out a multivariate analysis using logistic regression analysis. A *p* value of <0.05 was considered to be statistically significant.

### 2.9. Ethics Committee Approval

The research complies with the ethical standards for human experimentation, as indicated by the ethics committee of the Hospital Clínico Universitario Virgen de la Arrixaca with number NE-2020-5-HCUVA.

## 3. Results

### 3.1. Completation Rate

Of the 420 people selected, 84% (n = 351) completed the survey (Table 2). Sixty-nine people who refused to complete the survey were excluded for the following reasons: a lack of time, not wishing to talk about the topic or an assertive refusal.

### 3.2. General Variables about ODT

#### 3.2.1. Attitude toward Organ Donation

Fifty-three percent (n = 185) of the respondents would donate their own organs, 25% (n = 89) would not and 22% (n = 77) had doubts about it. The reasons provided for being in favour of donating one’s organs were mainly: solidarity (96%, n = 178), reciprocity (58%, n = 107), moral obligation (13%, n = 24), and religious reasons (9%, n = 16). The respondents who were not in favour of donating their own organs reported: fear of apparent death (37%, n = 62), rejection of the idea of body mutilation (24%, n = 40), being too old to be a donor (23%, n = 38), not wanting to give their reasons (21%, n = 35), disease (19%, n = 31), religious motives (2%, n = 4), and not wanting to talk about death (2%, n = 4).

#### 3.2.2. Social and Family Interaction Related to ODT

Most respondents had not spoken about ODT in their family setting (67%, n = 236) or with friends (79%, n = 276). Thirty-six percent (n = 125) knew that their partner had a favourable attitude toward ODT, 14% (n = 49) knew that it was unfavourable, and 48% (n = 170) of respondents did not know their partner’s attitude. 

#### 3.2.3. Awareness of Life Support Limitation and Attitude towards Controlled Donation after Circulatory Death

Nine percent (n = 33) of the respondents know the concept of life support limitation, 90% (n = 315) do not and 1% (n = 3) had doubts about it. On the other hand, 45% (n = 158) would be in favour about controlled donation after circulatory death, 29% (n = 103) would not and 26% (n = 90) had doubts about it.

### 3.3. Psychosocial Profile Related to Their Attitude toward the Donation of Their Own Organs upon Death

#### 3.3.1. Sociopersonal Variables

Sociopersonal variables did not affect attitude toward ODT (*p* > 0.05), as can be seen in Table 3.

#### 3.3.2. Variables of Knowledge about ODT

The variables of knowledge are shown in Table 2. Knowledge of the concept of BD does not have a significant influence on attitude toward the donation of one’s own organs (*p* > 0.05). However, respondents who had known a donor had a more favourable attitude toward donation than those who had not (74% versus 47%; *p* < 0.001).

Most respondents had not received a talk about ODT (95%, n = 332), but were not interested in receiving one either (77%, n = 271). Apart from that, a more favourable attitude to donation has been found among those who had received a talk about the subject (95% vs. 50%; *p* < 0.001) or who wanted to receive one (70% vs. 48%; *p* < 0.001).

#### 3.3.3. Variables of Social and Family Interaction about ODT

Those who had discussed the subject of ODT with their family had a more favourable attitude compared to those who had not (69% versus 45%; *p* < 0.001). The same occurred when dialogue about the subject was with friends (76% versus 46%; *p* < 0.001) (Table 2). What is more, those respondents whose partner had a favourable opinion about ODT had a more favourable attitude toward donation compared to those who had a partner with an unfavourable or unknown opinion (79%, 39%, 36%, respectively; *p* < 0.001) (Table 2).

#### 3.3.4. Variables of Attitude toward the Manipulation of the Body

If we consider the attitudinal variables related to manipulation of the body in Table 3, we can identify individuals who accept cremation and autopsy as having a more favourable attitude toward donation than those who would not accept these practices (70% vs. 45% and 65% vs. 37%, respectively; *p* < 0.001). As a further point, attitude was less favourable toward donation in the group that was in favour of burial (48% vs. 68%; *p* < 0.001). 

When there was worry about scars or mutilation after organ donation, a more unfavourable attitude was found (20% vs. 58%; *p* < 0.001) (Table 4).

#### 3.3.5. Variables of Prosocial Behaviour

The respondents who stated that they were usually or occasionally blood donors, or would be willing to donate blood, had a more favourable attitude toward donation than those who did not want to be donors (63%, 60%, 69% vs. 45%; *p* = 0.011). Alternatively, although attitude was slightly more favourable in people who participated in prosocial activities, no significant differences were found (*p* > 0.05) (Table 3).

#### 3.3.6. Variables of Religion

Most respondents were Catholics. Their religious status was not associated with attitude toward the donation of their own organs (*p* > 0.05). Knowing that their religion had a favourable position in terms of ODT did not affect attitude either (*p* > 0.05) (Table 5).

### 3.4. Multivariate Analysis

The variables that persisted as significant in the multivariate analysis were: having a discussion with friends about the subject of ODT Odds Ratio (OR 2.223); acceptance of cremation after death (OR 2.508); and acceptance of an autopsy after death (OR 2.578) (Table 4).

## 4. Discussion

The present study, which was carried out in our region with a representative sample of more than 400 citizens aged over 65 years, reflects how a favourable attitude toward donating one’s own organs after death reaches a percentage of only 53% of the respondents, similar to those results reported in other studies [8,9]. Population studies carried out previously have shown how this subgroup has a more unfavourable attitude than the general population [13], which has a favourable attitude of between 60% and 70% [8,9]. On the other hand, the favourable attitude towards controlled donation after circulatory death is even lower, reaching only 45% of respondents.

Solidarity and reciprocity are the main reasons that lead people to support organ donation [8,9,14]. Citizens who are older than 65 years who have a greater willingness to donate organs also state these reasons. These values have a greater presence in advanced ages due to the increase in the frequency of physical and mental conditions in this stage of life [15]. Nevertheless, reciprocity, the second reason given, was only provided by 56% of the respondents. This piece of data could be related to the fact that only 21% of respondents believe that they might need an organ at some future point in time. 

A fourth of the respondents had erroneous concepts about ODT, which could lead them to be less committed to the process, as suggested by other studies [13,14,16]. It has been seen that 23% of respondents believed they were too old to be a donor and 19% believed they were not able to be donors due to some kind of disease. Therefore, it is necessary to provide adequate information in this regard, stressing the fact that more than 50% of donors are older than 60 years and there are only a small number of contraindications due to disease that can prevent someone from being a donor [1]. On the other hand, knowledge of the limitation of life support and the possibility of controlled donation after circulatory death is another way of donation that has been on the rise in recent years [6] and which should be included in the information that should be provided to this group regarding organ donation.

It has also been seen that only 2% indicated that they did not want to talk about death. Given the advanced age of some of the respondents, this has not been as much of a taboo topic for respondents as it was in other studies [13,17]. In this sense, some recent studies have shown that the subject of death is not seen as a taboo topic in discussions held by older people, in spite of being conditioned by their clinical situation, culture and religious beliefs [18].

The reasons for being against organ donation given most by the respondents were fear of apparent death and the rejection of the idea of mutilation of the body, as seen in other studies [8,9]. The misconception of the BD concept reached 36% of the respondents of older age in our region [19], although knowledge of this concept did not significantly influence attitude toward the donation of their own organs, which is something that does occur in the general population and in other groups [19,20].

Having previous experience of the ODT process is also important. Contact with the donor, a transplant patient, or someone who needs a transplant teaches us about the reality of ODT and encourages values such as solidarity, which motivate people to have a more favourable attitude [8,9]. In our study, it has been seen how knowing a donor significantly influences older people’s attitudes toward the donation of their own organs. This finding could be a crucial indication that innovative educational intervention strategies should be promoted in this group, especially taking into account the high percentage of respondents who rejected the idea of receiving a talk about the subject.

It has been seen how variables of social and family interaction have had the most influence on the attitude of older people toward the donation of their own organs, together with the variables of attitude toward the body after death.

Recently, we published a study concerning the means of information through which older people in our region received information about ODT [21]. In this study, it was seen how older people mainly receive information about ODT from the mass media (television, films, and radio). However, social and family circles have the greatest influence on their attitudes toward organ donation [21]. 

Only 20–30% of the respondents indicated that they had discussed the subject of ODT in a social or family setting. However, it has been seen how social and family discussions, and knowing the opinion of one’s partner, both have an influence on attitude toward the donation of one’s own organs, with similar findings reported in other studies [22,23,24]. Family discussion about ODT not only makes it possible to keep families informed about each family member’s ideas, but it also provides an opportunity to talk about the information obtained through other means, personal opinions, and fears about the topic; and it produces an exchange of knowledge [25].

Normally, families respect the wishes of the deceased when these have been expressed [9]. Therefore, it is very important for people who are in favour of donating their organs to communicate their wishes to family members, taking into account the presumed consent law established in Spain in which the family decision is taken into account [26,27]. When a family is faced with the donation of a family member’s organs, they find themselves at a time of mourning when making this decision is complicated [28]. In this sense, when the family knows the wishes of the deceased, then this relieves the stress brought about by having to decide whether to donate their organs. Generally, the wishes of the deceased person constitute the most important predictor of the family decision [29], which is why it is important to emphasize the need for social and family dialogue about ODT in older people.

Attitude toward the treatment of the body after death is influenced by the ideas someone has about what death is like, how it happens… which means that this is a relevant factor affecting attitude toward the donation of one’s own organs [8,9,16,30,31,32]. In the present study, acceptance of cremation was found in only 31% of the respondents, but is associated with a better attitude toward the donation of one’s own organs, persisting as a significant variable in the multivariate analysis alongside acceptance of an autopsy. On the other hand, the respondents who were worried about scars or mutilation of the body were less inclined to donate their organs, and this fact could explain why these people tend to opt to a greater extent for burial and the rejection of autopsy. 

Our data reflect the fact that attitude toward the donation of one’s own organs is not affected by the religion that a person follows, or knowing the position of one’s religion with regard to this matter. Despite most religions not being against ODT, including Catholicism, in many studies, religion has been a negative influence on attitude and the decision to donate [8,9,25,30,33].

### Limitations of the Study

Despite the interesting results obtained, it would be important to carry out this type of study in other regions to detect other possible factors that may influence the predisposition towards organ donation in the elderly according to cultural or racial differences in other countries where organ donation rates are lower.

## 5. Conclusions

Citizens over the age of 65 years in southeastern Spain tend to have an unfavourable attitude toward the donation of their own organs upon death. The aspects of the psychosocial profile that have an influence on this attitude are fundamentally shaped by the lack of social and family discussion about ODT and attitude toward the manipulation of one’s own body after death. These data reflect the most important aspects to be taken into account in order to design new educational intervention strategies to improve knowledge about ODT and willingness to donate organs in citizens of an older age.

## Figures and Tables

**Table 1:** Validated questionnaire about attitude toward ODT.

Sociopersonal Information	Options
Age	65–70 years 71–75 years 76–80 years >80 years
Gender	Male Female
Marital Status	Single Married or with a partner Separated/divorced Widowed
**Knowledge About ODT**	
Do you know any organ donors?	Yes No
Do you know any transplant patients?	Yes No
Do you think you might need a transplant in the future?	Yes No DS/DK
Have you received a talk about ODT?	Yes No
Would you like to receive a talk about ODT?	Yes No
Can a person with brain death recover and live a normal life?	Yes No DS/DK
Are you familiar with the term life-sustaining treatment limitation?	Yes No DS/DK
If this were the case, would you be in favour of controlled donationafter circulatory death, considered after the limitation of life-sustaining treatment?	Yes No DS/DK
**Social and family interaction**	
What do you believe is the position of your religion toward ODT?	In favour Against DS/DK
Have you discussed the subject of organ donation and transplantationwith your family?	Yes No
Have you discussed the subject of organ donation and transplantationwith your friends?	Yes No
Do you know your partner’s opinion about organ donation?	Yes. It is or was favourable. I do not or did not know it. Yes. He or she is or was against it. DS/DK
**Attitude toward manipulation of the body**	
Are you in favour of cremation after death?	Yes No DS/DK
What is your opinion on burial after death?	Yes No DS/DK
What is your opinion on doing an autopsy after death?	Yes No DS/DK
What is your opinion on scarring or mutilation of the body after death?	Yes No DS/DK

**Table 2:** Questionnaire completion rate of the study population stratified by gender and geographical location.

	Male		Female		Total	
Area	N0	N1	N0	N1	N0	N1
1. Jumilla–Yecla area	5 (45%)	4 (50%)	6 (55%)	4 (50%)	11 (3%)	8 (2%)
2. Lorca area	12 (43%)	11 (44%)	16 (57%)	14 (56%)	28 (7%)	25 (7%)
3. Bajo Guadalentín	9 (47%)	9 (50%)	10 (53%)	9 (50%)	19 (5%)	18 (5%)
4. Campo de Cartagena–Mar Menor	30 (43%)	34 (46%)	39 (57%)	40 (54%)	69 (16%)	74 (21%)
5. Murcia Metropolitan area	116 (47%)	84 (47%)	130 (53%)	93 (53%)	246 (58%)	177 (51%)
6. Northwest	8 (44%)	7 (37%)	10 (56%)	12 (63%)	18 (4%)	19 (5%)
7. Abanilla–Fortuna area	2 (50%)	1 (50%)	2 (50%)	1 (50%)	4 (1%)	2 (1%)
8. Mula area	2 (40%)	4 (44%)	3 (60%)	5 (56%)	5 (1%)	9 (3%)
9. Vega del Segura	9 (45%)	7 (37%)	11 (55%)	12 (63%)	20 (5%)	19 (5%)
Total	193 (46%)	161 (46%)	227 (54%)	190 (54%)	420 (100%)	351 (100%)

N0: Sample size. N1: Sample obtained.

**Table 3:** Analysis of the influence of the sociopersonal variables on attitude toward the donation of one’s own organs upon death and of the variables of knowledge and social/family interaction about ODT on attitude to the donation of one’s own organs upon death.

Sociopersonal Variables	In Favour	Not in Favour	*p* Value	Odds Ratio (Confidence Interval)	*p* Adjusted Value
	(n = 185; 53%)	(n = 166; 47%)			
Age					
65–70 years (n = 162; 46%)	96 (59%)	66 (41%)	0.109	0.004 (−0.005; 0.012)	0.386
71–75 years (n = 93; 27%)	47 (51%)	46 (49%)			
76–80 years (n = 57; 16%)	25 (44%)	32 (56%)			
>80 years (n = 39; 11%)	17 (44%)	22 (56%)			
Area					0.605
Jumilla–Yecla area (n = 8; 2%)	6 (75%)	2 (25%)	0.303	−0.008 (−0.039; 0.023)	
Lorca area (n = 25; 7%)	15 (60%)	10 (40%)			
Bajo Guadalentín (n = 18; 5%)	9 (50%)	9 (50%)			
Campo Cartagena–Mar Menor (n = 74; 21%)	41 (55%)	33 (45%)			
Metropolitan area of Murcia (n = 177; 50%)	87 (49%)	90 (51%)			
Northwest (n = 19; 5%)	8 (42%)	11 (58%)			
Abanilla–Fortuna area (n = 2; 1%)	0 (0%)	2 (100%)			
Mula area (n = 9; 3%)	7 (78%)	2 (22%)			
Vega del Segura (n = 19; 5%)	12 (63%)	7(37%)			
Gender					0.582
Male (n = 161; 46%)	85 (53%)	76 (47%)	0.976	−0.03 (−0.138; 0.077)	
Female (n = 190; 54%)	100 (53%)	90 (47%)			
Marital status					
Single (n = 15; 4%)	8 (53%)	7 (47%)	0.553	0.001 (−0.004; 0.045)	0.973
Married or with a partner (n = 269; 77%)	143 (53%)	126 (47%)			
Separated/divorced (n = 15; 4%)	10 (67%)	5 (33%)			
Widowed (n = 52; 15%)	24 (46%)	28 (54%)			
**Variables of knowledge about ODT**					
Knowledge of the concept of BD			0.756		
Erroneous concept (n = 104; 30%)	52 (50%)	52 (50%)		−0.019 (−0.081; 0.042)	0.541
Correct concept (n = 127; 36%)	67 (53%)	60 (47%)			
Not known (n = 120; 34%)	66 (55%)	54 (45%)			
Knowing a donor					
Yes (n = 72; 21%)	53 (74%)	19 (26%)	<0.001	0.125 (−0.009; 0.258)	0.067
No (n = 279; 79%)	132 (47%)	147 (53%)			
Knowing a transplant patient					
Yes (145; 41%)	83 (57%)	62 (43%)	0.153	−0.03 (−0.134; 0.074)	0.574
No (279; 60%)	102 (50%)	104 (50%)			
Believing that one might need a transplant in the future					
Yes (n = 72; 21%)	41 (57%)	31 (43%)	0.638	0.007 (−0.056; 0.069)	0.835
No (n = 116; 33%)	62 (53%)	54 (47%)			
Not known (n = 163; 46%)	82 (50%)	81 (50%)			
Have you received a talk about ODT?					
Yes (n = 19; 5%)	18 (95%)	1 (5%)	<0.001	0.179 (−0.047; 0.404)	0.120
No (n = 332; 95%)	167 (50%)	165 (50%)			
Would you like to receive a talk about ODT?					
Yes (n = 80. 23%)	56 (70%)	24 (30%)	<0.001	0.179 (−0.047; 0.404)	0.008
No (n = 271; 77%)	129 (48%)	142 (52%)			
**Variables of social and family interaction**					
You have spoken about ODT with your family					
Yes (n = 115; 33%)	79 (69%)	36 (31%)	<0.001	−0.005 (−0.136; 0.125)	0.934
No (n = 236; 67%)	106 (45%)	130 (55%)			
You have spoken about ODT with your friends					
Yes (n = 75; 21%)	57 (76%)	18 (24%)	<0.001	0.079 (−0.06; 0.218)	0.266
No (n = 276; 79%)	128 (46%)	148 (54%)			
Your partner’s opinion about ODT					
Yes. It is or was favourable. (n = 125; 36%)	99 (79%)	26 (19%)	<0.001	0.099 (0.033; 0.165)	0.004
I do not or did not know it. (n = 170; 48%)	62 (36%)	108 (64%)			
Yes. He or she is or was against it. (n = 49; 14%)	19 (39%)	30 (61%)			
DS/DK (n = 7; 2%)	5	2			

*p* < 0.05: statistically significant.

**Table 4:** Analysis of the influence of variables of attitude toward the manipulation of the body, of prosocial behaviour and religion, on attitude toward organ donation upon death.

Variables of Attitude toward Manipulation of the Body	In Favour	Not in Favour	*p* Value	Odds Ratio (Confidence Interval)	*p* Adjusted Value
	(n = 185; 53%)	(n = 166; 47%)			
Acceptance of cremation					
Yes (n = 108; 31%)	76 (70%)	32 (30%)	<0.001	0.103 (−0.051; 0.257)	0.191
No (n = 242; 69%)	108 (45%)	134 (55%)			
* DK/NS (n = 1; 0%)*	1	0			
Acceptance of burial					
Yes (n = 108; 31%)	124 (48%)	137 (52%)	<0.001	−0.034 (−0.188; 0.12)	0.664
No (n = 242; 69%)	60 (68%)	28 (32%)			
* DK/NS (n = 2; 1%)*	1	1			
Acceptance of an autopsy					
Yes (n = 201; 57%)	130 (65%)	71 (35%)	<0.001	0.163 (0.58; 0.267)	0.002
No (n = 150; 43%)	55 (37%)	95 (63%)			
Worry about scars or mutilation after organ donation					
Yes. It worried me a lot (n = 41; 12%)	8 (20%)	33 (80%)	<0.001	−0.129 (−0.271; 0.012)	0.072
I do not mind (n = 304; 86%)	175 (58%)	129 (42%)			
I do not know (n = 6; 2%)	2 (33%)	4 (67%)			
**Variables of prosocial behaviour**					
Being a blood donor			0.011		
Yes, normally (n = 27; 8%)	19 (63%)	11 (37%)			
Yes, occasionally, or I gave blood once (n = 78; 24%)	50 (60%)	33 (40%)		−0.001 (−0.051; 0.049)	0.980
No, but I would be willing to (n = 34; 10%)	24 (69%)	11 (31%)			
No, and I will not be one (n = 190; 58%)	92 (45%)	111 (55%)			
Carrying out prosocial activities					
Yes, normally (n = 126; 36%)	76 (60%)	50 (40%)	0.071	−0.04 (−0.094; 0.014)	0.150
Yes, occasionally (n = 89; 25%)	40 (45%)	49 (55%)			
No, nor will I (n = 114; 33%)	55 (48%)	59 (52%)			
No, but I would be willing to (n = 22; 6%)	14 (64%)	8 (36%)			
**Variables of social and family interaction**					
Religious attitude					
Practising Catholic (n = 145; 41%)	67 (46%)	78 (54%)	0.178	−0.07 (−0.142; 0.002)	0.056
Non-practising Catholic (n = 186; 53%)	105 (57%)	81 (43%)			
Non-Catholic religion (n = 4; 1%)	3 (75%)	1 (25%)			
Agnostic—atheist (n = 16; 5%)	10 (63%)	6 (37%)			
What do you believe is the position of your religion toward ODT?					
It is in favour (n = 132; 38%)	69 (52%)	63 (48%)	0.991	−0.003 (−0.038; 0.032)	0.853
It is against (n = 38; 11%)	20 (53%)	18 (47%)			
I do not know (n = 181; 51%)	96 (53%)	85 (47%)			

*p* < 0.05: statistically significant.

**Table 5:** Multivariate analysis.

Variable	Regression Coefficient (β)	Standard Error	Odds Ratio (Confidence Interval)	*p* Value
You have discussed ODT with your friends	0.799	0.336	2.223 (1.150–4.298)	0.017
Acceptance of cremation	0.920	0.286	2.508 (1.433–4.392)	0.001
Acceptance of an autopsy	0.947	0.263	2.578 (1.540–4.318)	<0.001

*p* < 0.05: statistically significant.

## Data Availability

Data are contained within the article.
